# Search for new α1a-adrenoceptor-selective antagonist for treating lower urinary tract symptoms associated with benign prostatic hyperplasia

**Published:** 2007

**Authors:** Naval Khurana, Aneesh Srivastava

**Affiliations:** Department of Urology and Renal Transplant, SGPGIMS, Lucknow, India. E-mail: drrgoyal@yahoo.com

## SUMMARY

This phase III randomized, multicentric, double-blind, placebo-controlled, study[1] in Japanese men has shown the clinical efficacy of silodosin, a new α_1A_ -adrenoceptor-selective antagonist for treating lower urinary tract symptoms (LUTS) associated with benign prostatic hyperplasia (BPH). The change in the total International prostatic symptom score (IPSS) from baseline in the silodosin, tamsulosin and placebo groups was −8.3, −6.8 and −5.3, respectively. There was a significant decrease in the IPSS vs. placebo in the silodosin group from one week. In the early-stage comparison, silodosin showed a significant decrease in IPSS vs. tamsulosin at two weeks. The change in QoL from baseline was −1.7, −1.4 and −1.1 in the silodosin, tamsulosin and placebo groups, respectively. Silodosin showed a significant improvement in the QoL score vs. placebo. In the subgroup of patients with severe symptoms (IPSS ≥ 20) silodosin also gave a significantly better improvement than placebo (−12.4 vs. −8.7). The incidence rates of adverse events and drug-related adverse events were, respectively, 88.6%, 82.3% and 71.6% and 69.7%, 47.4% and 36.4%, respectively. The most common adverse event in the silodosin group was abnormal ejaculation, which occurred more often in the silodosin than in the tamsulosin group (22.3% vs. 1.6%).

## COMMENTS

It is well established that all α_1_ -adrenoceptor blockers currently recommended for treating LUTS (alfuzosin, doxazosin, tamsulosin, terazosin) have equal clinical effectiveness, producing a mean 4-6 point improvement in the IPSS.[2] However, they differ in side-effect profiles due to difference in the affinity of these blockers on α-adrenoceptor. In the AUA meta-analysis,[2] while the four α1 -adrenoceptor blockers showed incidences of decreased libido (1-3%) and erectile dysfunction (3-5%) closely similar to placebo (3 and 4% respectively), tamsulosin was associated with higher incidence of ejaculatory dysfunction (10%) than other α_1_ -adrenoceptor blockers (0-1%) and placebo (1%).The incidence of ejaculatory dysfunction with tamsulosin not only increases with dose[3] but also increases with time.[4]

To date several α_1_ -adrenoceptor blockers with very high α_1_ -adrenoceptor selectivity have been developed, but their efficacy and safety profiles have not been confirmed clinically in patients with BPH. This study[1] has shown better efficacy of silodosin over tamsulosin (0.2 mg) with more incidence of ejaculatory dysfunction (22.3% vs. 1.6%) but only 2.9% discontinuation rate. Better efficacy might elicit improvement even in patients who could be candidates for surgical therapy but at the cost of more side-effects. Better effect of silodosin could be due to high selectivity for α_1a_ -adrenoceptor or due to suboptimal does of tamsulosin (0.2 mg) even in the apparently ‘α blocker- ultrasensitive’ Japanese population.

When treating LUTS associated with BPH it is necessary to focus not just on symptoms generated by bladder outlet obstruction (voiding symptoms mediated by α_1a_-adrenoceptor) but also on symptoms generated by bladder dysfunction (storage symptoms mediated by α_1d_-adrenoceptor). Both silodosin and tamsulosin have more affinity for α_1a_ -adrenoceptor hence are more effective in relieving voiding symptoms. Drugs alleviating both storage and voiding symptoms have been discovered like naftopidil which has high affinity for α_1d_[5] in comparison to α_1a_-adrenoceptor. Naftopidil has been found more effective than tamsulosin in alleviating storage symptoms with equal efficacy on voiding symptoms.[6] Hence search for potent and selective α1- adrenoceptor blockers with minimal side-effects for treatment of LUTS associated with BPH is still continuing.
